# Evaluation of an international medical E-learning course with natural language processing and machine learning

**DOI:** 10.1186/s12909-021-02609-8

**Published:** 2021-03-25

**Authors:** Aditya Borakati

**Affiliations:** University Department of Surgery, Royal Free Hospital, Pond Street, London, NW3 2QG UK

**Keywords:** Methods, Research design, Machine learning, Education, Computer-assisted instruction

## Abstract

**Background:**

In the context of the ongoing pandemic, e-learning has become essential to maintain existing medical educational programmes. Evaluation of such courses has thus far been on a small scale at single institutions. Further, systematic appraisal of the large volume of qualitative feedback generated by massive online e-learning courses manually is time consuming. This study aimed to evaluate the impact of an e-learning course targeting medical students collaborating in an international cohort study, with semi-automated analysis of feedback using text mining and machine learning methods.

**Method:**

This study was based on a multi-centre cohort study exploring gastrointestinal recovery following elective colorectal surgery. Collaborators were invited to complete a series of e-learning modules on key aspects of the study and complete a feedback questionnaire on the modules. Quantitative data were analysed using simple descriptive statistics. Qualitative data were analysed using text mining with most frequent words, sentiment analysis with the AFINN-111 and syuzhet lexicons and topic modelling using the Latent Dirichlet Allocation (LDA).

**Results:**

One thousand six hundred and eleventh collaborators from 24 countries completed the e-learning course; 1396 (86.7%) were medical students; 1067 (66.2%) entered feedback. 1031 (96.6%) rated the quality of the course a 4/5 or higher (mean 4.56; SD 0.58). The mean sentiment score using the AFINN was + 1.54/5 (5: most positive; SD 1.19) and + 0.287/1 (1: most positive; SD 0.390) using syuzhet. LDA generated topics consolidated into the themes: (1) ease of use, (2) conciseness and (3) interactivity.

**Conclusions:**

E-learning can have high user satisfaction for training investigators of clinical studies and medical students. Natural language processing may be beneficial in analysis of large scale educational courses.

**Supplementary Information:**

The online version contains supplementary material available at 10.1186/s12909-021-02609-8.

## Background

E-learning is ubiquitous worldwide in both undergraduate [1, 2] and postgraduate medical education [3]. Diverse topics ranging from anatomy [4] to evidence based medicine [5] and clinical research training [6] may be delivered by e-learning. These courses may be delivered by single institutions to discrete cohorts or asynchronously to any number of students worldwide as in Massively Open Online Courses (MOOCs [7]).

These courses have been shown to have similar student satisfaction to traditional, face-to-face instruction [5, 8–11], whilst being significantly cheaper to run [12, 13].

Further, in the context of the ongoing COVID-19 pandemic, with nationwide lockdowns and social distancing measures precluding face-to-face instruction in many cases, e-learning has become essential. Many medical educational programmes have shifted to e-learning due to the pandemic [14, 15].

Evaluating large volumes of qualitative data that these large online courses generate is a further challenge. Natural language processing (NLP) and machine learning aggregation methods are common in commercial contexts on the internet.

The key methods in NLP are sentiment analysis and topic modelling. The former involves assigning each word in a body of text a numeric value, typically representing positivity or negativity based on pre-existing dictionaries (known as lexicons) which have already assigned every word a value for positivity or negativity. The values are averaged across the whole body of text to give an overall value of sentiment for the text. Topic modelling involves using machine learning algorithms to automatically group related words into relevant topics or themes.

These methods have been used in educational studies generally to gather overall sentiment [16–18] of bodies of free text and to generate overarching topics or themes from this [19]. However, these methods have yet to be utilised in medical education contexts [20, 21].

In this article, the experience of deploying a mandatory e-learning course for investigators (the majority of whom were medical students in clinical training) in an international multicentre collaborative cohort study, the IMAGINE study [22] is reported. This study aimed to evaluate the experience of course participants and identify key themes for future development of e-learning courses using NLP and machine learning methods and a guide is provided for other researchers to implement this in their own work.

## Materials and methods

### Research platform

The Ileus Management International (IMAGINE) study was a multinational collaborative study assessing gastrointestinal recovery following elective colorectal surgery. It took place between January and March 2018 at 424 hospitals across 24 countries in Europe and Australasia. Collaborators must have been a medical student in the clinical years of their course or a qualified doctor to participate [22, 23].

### Module development

A series of four e-learning modules were developed for investigators in the study, covering four key phases of protocol implementation: (1) Study eligibility criteria; (2) Outcome assessment; (3) Data collection procedures; (4) Data security (Table 1). Content was agreed by the study steering group to ensure consistency with the final study protocol. Modules were developed using the H5P software (H5P, Joubel AS, Tromsø, Norway) on Wordpress 4.7.4 (Wordpress, Automattic Inc., San Francisco, CA, USA). The e-learning modules included multimedia, interactive content and multiple-choice questions based on clinical vignettes to assess learning. Learning objectives were written based on Bloom’s taxonomy. Modules were designed to take less than 30 min to complete.

**Table 1:** Summary of e-learning modules; modules are accessible at: https://starsurg.org/imagine-e-learning/

Module Name	LearniLearning Objectives
IMAGINE: Included Patients and How to Find Them	○ Know the inclusion and exclusion criteria for the IMAGINE project ○ Be able to apply them to patients for the IMAGINE project, correctly identifying which patients need including and excluding
Gastrointestinal Function	○ Have an understanding of the GI-2 measure for gastrointestinal recovery ○ Know the components assessed for gastrointestinal function ○ Be able to find the relevant data for gastrointestinal function in IMAGINE
The Clavien-Dindo Classification	○ Understand the Clavien-Dindo classification and its importance ○ Know the different grades of the Clavien-Dindo classification ○ Find the relevant information you need in the clinical setting to assign a Clavien-Dindo grade ○ Apply the Clavien-Dindo classification to patients post-operatively, giving them an accurate score
REDCap and Data Safety	○ Be able to keep patient data safe and secure at your hospital site ○ Know how to login to the REDCap server for data entry to the IMAGINE project ○ Know how to enter data for the IMAGINE project

Modules were publicly accessible from a web browser and could be translated to the users’ language using an embedded Google Translate widget (Google Translate, Alphabet Inc., Mountain View, CA, USA) from English [24]. Translated modules were reviewed by native speakers in the respective languages for accuracy; if there were significant inaccuracies, users were advised to complete the modules in English. A basic level of English language proficiency was required for participation in the study and all translations could be reverted into the original English by hovering over selected text to ensure consistency. A disclaimer was given to check the native English before completing the module.

### Participant evaluation

After completion of the modules, investigators were invited to complete an anonymous, voluntary online feedback questionnaire (Google Forms, Alphabet Inc., Mountain View, CA, USA; Table 2, supplemental file 2 and [24]). This was a closed survey accessible only to course participants and was available at the end of course. Feedback was only allowed to be entered in English. Participation was voluntary with no incentivisation. Respondents were only permitted to complete one entry and this was enforced by evaluating the unique cookie for each user. Users were able to submit feedback for analysis at any time following completion of the course between October 2017 to June 2018. Revision of responses was not allowed.

**Table 2:** Feedback questionnaire items; *indicates mandatory question; questionnaire accessible at: https://starsurg.org/imagine-e-learning/ (no longer taking responses)

Question	Response options
(1) How would you rate the e-learning overall?*	Very bad 1 2 3 4 5 Very good
**(2) What was good about the e-learning overall?**	**Free text**
**(3) What could be improved about the e-learning overall?**	**Free text**
**(4) Any other comments about the e-learning overall?**	**Free text**
**(5) Add any specific comments about module 1 (Inclusion/Exclusion criteria) here:**	**Free text**
**(6) Add any specific comments about module 2 (Gastrointestinal Function) here:**	**Free text**
**(7) Add any specific comments about module 3 (Clavien-Dindo Classification) here:**	**Free text**
**(8) Add any specific comments about module 4 (REDCap and Data Protection) here:**	**Free text**

The questionnaire contained both quantitative (Likert scale) and qualitative (free text) where investigators could comment on the quality and utility of the course and its individual modules. All items were displayed on one single page.

Feedback was periodically reviewed during the time period that the e-learning course was live and minor corrections (e.g. grammar, spelling) were made as appropriate in response. Satisfactory completion of the modules, with a 75% pass mark, was mandatory for participation in the study. 75% was chosen as a pragmatic threshold, which allowed a reasonable degree of understanding, whilst not being too arduous to complete. Participants could repeat each question as many times as they required to pass.

### Analysis

Data was analysed using Microsoft R Open 3.5.3 for Windows (Microsoft Corporation, Redmond, WA, USA). Analysis scripts and a guide on implementing the methods discussed here is available in supplementary file 1 and online [25].

Distributions of continuous data was assessed by visual inspection of histograms, those following a normal distribution were presented using means with standard deviations or percentages. Non-normally distributed data were summarised as medians and interquartile range.

Qualitative analysis of the free text responses was conducted according to a content analysis framework, using NLP and machine learning techniques as described below.

Free text data was coded using a text mining method with the ‘tm’ R package [26]. Responses were merged into a single body of text (corpus) for each question and each word ranked by frequency. Common English ‘stop words,’ such as ‘the’ and ‘a’ were then removed using the ‘stopwords’ function in tm. The resulting corpus left only key adjectives for analysis of the responses. These are presented below and divided into positives and negatives based on the question asked in the questionnaire.

An unsupervised machine learning approach (Latent Dirichlet Allocation (LDA) algorithm with Gibbs sampling [27, 28]) was used to generate overarching topics in the free text. The number of topics to be generated was determined by calculating the metrics for each number of topics using four different formulae [29–32] and then finding the optimum number of topics by combined graphical analysis of these functions as described by Nikita [33]. Further evaluation of the topics generated by this algorithm is described in supplementary file 1 with an explanation of the metrics.

Sentiment analysis was conducted with all free text merged (without stop words) into one corpus. Each word was assigned a sentiment score using both the AFINN-111 [34] and syuzhet [35] lexicons, two commonly used general purpose lexicons which assign each word in the dictionary a rating of positivity or negativity. The mean score in each lexicon was calculated for the whole corpus to give a quantitative measure of the overall positivity or negativity of the free text feedback. Valence shifters in sentences e.g. ‘this was not good’ which may have the sentiments incorrectly calculated by the lexicons were accounted for as described in the sentimentr package [36].

**Fig. 1:**
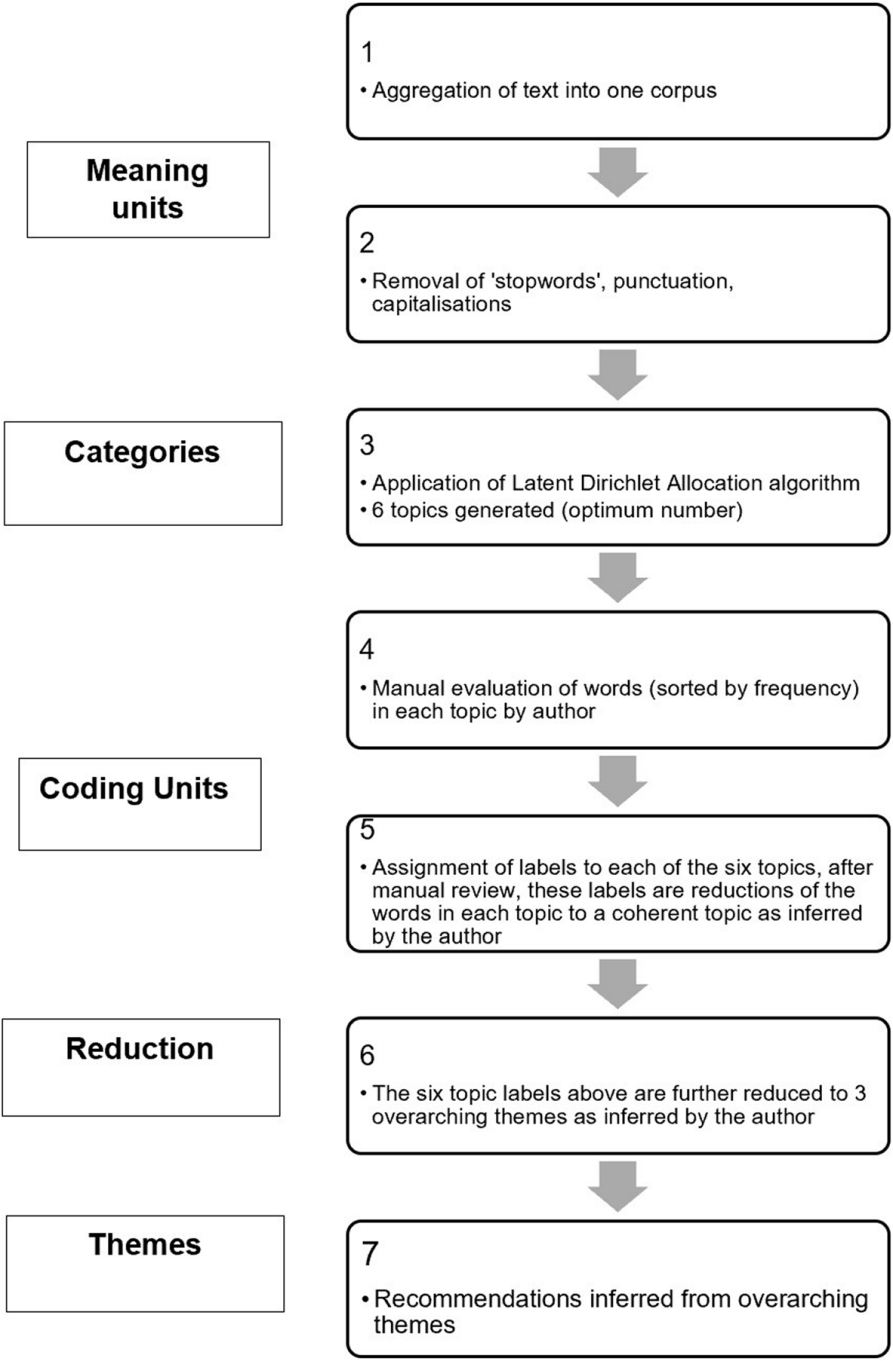
Workflow for natural language processing and machine learning assisted content thematic analysis; note analogies to steps in traditional content analysis framework

Overall consolidated thematic analysis of the free text including recommendations for future international research projects was then conducted by reviewing the results of the three analyses above, this was performed by one author (AB). The workflow for the qualitative analysis is shown in Fig. 1.

### Ethics & Governance

Data was stored in accordance with European Union General Data Protection Regulations and users were informed that their anonymous feedback may be used in research and improvement of future courses as per the privacy policy as a condition of participation [37]. As such implicit informed consent was obtained from all subjects. No formal ethics committee approval was sought for this study as only anonymised feedback data was collected.

This article is reported according to the SRQR guidelines for qualitative research [38] and the CHERRIES guidelines for online surveys, [39] endorsed by the EQUATOR network [40].

## Results

Overall, 1611 collaborators attempted the e-learning course, of whom 1067 (66.2%) entered feedback. The demographics of the participants are summarised in Table 3: the majority (1396; 86.7%) were medical students; 200 (12.4%) were junior doctors, and 15 (0.9%) were consultant/attending surgeons.

**Table 3:** Demographics of e-learning participants

Total number completing course (n)	1611
Medical students (%)	1396 (86.7)
Junior doctors (%)	200 (12.4)
Consultants (%)	15 (0.9)
**Country**	
United Kingdom (%)	918 (57.0)
Australia/ New Zealand (%)	119 (7.4)
Spain (%)	106 (6.6)
Italy (%)	64 (4.0)
Republic of Ireland (%)	62 (3.8)
Other (%)	342 (21.2)
**Number completing feedback (%)**	**1067 (66.2)**

### Quantitative results

**Fig. 2:**
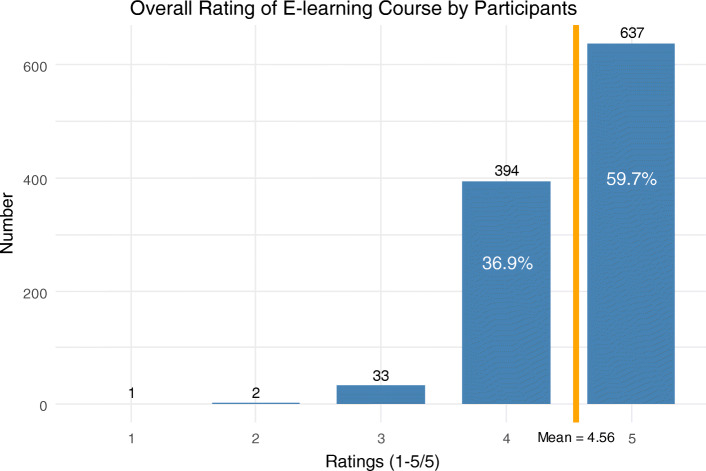
Responses to question ‘How would you rate the e-learning overall?’; 1 = ‘Very Bad,’ 5 = ‘Very Good,’ *n* = 1067

The mean of responses to the question ‘How would you rate the e-learning overall?’ was 4.56 out of 5 (Standard deviation (SD) 0.58). 637 (59.7%) respondents rated the course 5/5 (Fig. 2).

### Qualitative feedback

The feedback entered for the question ‘What was good about the e-learning overall?’ is shown Table 4. The three most common words entered were ‘easy,’ ‘clear’ and ‘concise.’ ‘Easy,’ was typically followed with words to the effect of ‘easy to follow.’

**Table 4:** Frequency of top 20 words entered in response to question ‘What was good about the e-learning overall?,’ *n* = 630 (59.0%)

Word	Frequency
Easy	125
Clear	93
Concise	79
Good	65
Information	58
Simple	55
Questions	45
Follow	40
Informative	39
Understand	37
Use	36
Interactive	30
Quick	28
Short	28
Well	23
Useful	20
Knowledge	19
Point	19
Cases	18

Feedback entered for the question ‘What could be improved about the e-learning overall?’ is shown in Table 5. The most common words were ‘nothing,’ ‘questions’ and ‘nil,’ with ‘questions’ typically being preceded by ‘more.’

**Table 5:** Frequency of top 20 words entered in response to question ‘What could be improved about the e-learning overall?,’ *n* = 426 (39.9%)

Word	Frequency
Nothing	68
Questions	37
Nil	17
Module	17
Redcap	15
Data	14
Videos	13
Information	12
Cases	11
Better	11
Examples	11
Can	11
Video	10
Spelling	10
Maybe	10
Good	10
None	8
Certificate	8
Test	7
Make	7

The 8 most common words entered for the section ‘Any other comments about the e-learning overall’ are shown in Table 6. The three most common were ‘good,’ ‘none’ and ‘nil.’ All other words had less than 5 entries.

**Table 6:** Frequency of top 8 words entered in response to question ‘Any other comments about the e-learning overall,’ *n* = 243 (22.8%)

Word	Frequency
Good	32
None	16
Nil	12
Easy	8
Well	7
Great	6
Overall	5

### Topic modelling

The optimum number of topics for LDA was calculated to be 6. The most common relevant words in each topic are shown in Table 7 with the suggested themes: ‘ease,’ ‘assessment,’ ‘comprehension,’ ‘interactivity,’ ‘clarity’ and ‘summary.’

**Table 7:** Selection of most common, relevant terms in topics generated from Latent Dirichlet Allocation topic modelling of combined free text feedback

Topics					
1	2	3	4	5	6
**Inferred Themes**					
**Ease**	**Assessment**	**Comprehension**	**Interactivity**	**Clarity**	**Summary**
Easy	Questions	Useful	Informative	Concise	Good
Knowledge	Tests	Simple	Short	Information	Clear
Information	Certificate	Examples	Interactive	Point	Quick
Study	Test	Explained	Cases	Clear	Summary
Clear	Quiz	Understand	Clinical	Succinct	Relevant

### Sentiment analysis

The mean sentiment score using the AFINN lexicon was + 1.54/5 (SD 1.19) and + 0.287/1 (SD 0.390) using the syuzhet lexicon for all free text feedback. These both indicated overall positive feedback which is congruent with the most common words in the positive feedback and negative feedback questions (Tables 4 and 5) which many reported had nothing negative to feedback.

### Thematic analysis

The overarching, consolidated themes identified were: (1) the ease of use of the modules, (2) succinctness and simplicity to understand the content, (3) interactivity with use of assessment and clinical vignettes.

### Reflections for application to future research

#### (1) Ease of use of the modules.

The modules were noted to be easy to use, with clear instructions on navigation and successful completion. Log in and registration processes were also mentioned to be simple with no email confirmation necessary.

The software used (H5P) further enabled straightforward navigation, accessibility and speed. Consequently, the modules generated were HTML5 packages, which allowed significantly better performance and mobile accessibility unlike many e-learning courses which depend on older Flash technologies. Navigation of the overall course was also fast with one single page for the whole course, reducing user frustration. In addition, course participants appreciated the ability to complete the course flexibly and return with their progress saved.

### Recommendation: design of e-learning should consider the technologies used and prioritise user friendly and responsive software

#### (2) Succinctness and simplicity to understand the content.

The modules each had clear learning objectives based on Bloom’s taxonomy [41]; text in each module was limited to small bullet points and relevant words highlighted in bold or in coloured boxes. Content was also varied with the use of images, diagrams and videos to convey information.

The overall length of the course was short and appreciated by participants. Although the time taken to complete it was not tracked, many responses found that it took less than 30 min to complete. The ability to come back and complete the course in discrete sittings further enhanced this benefit.

### Recommendation: concise modules with short, to the point sentences conveying key points are needed; the overall length of the course should be minimised

#### (3) Interactivity with use of assessment and clinical vignettes.

The interactive elements in the modules were positively received by participants. A range of elements were employed including true/ false questions, matching and multiple choice questions. These were interspersed throughout the modules but also at the end as a formal assessment.

The assessment questions were based around clinical vignettes that were likely to be encountered in the study as well as clinical practice. Collaborators found these helpful and practical for the study.

### Recommendation: ensure modules contain interactive elements throughout; ensure content is relevant to practice, clinical vignettes are recommended

## Discussion

This study showed that a massive online investigator training resource using e-learning was a successful, positively received modality for training collaborators, in particular, medical students, to deliver the protocol for this international study. The majority of these investigators were medical students and this shows the applicability of such courses to medical education in general.

This study was evaluated using online feedback questionnaires and analysed this large volume of data using natural language processing and machine learning techniques. To our knowledge, this is the only evaluation of an educational course in the medical field or in clinical studies to use such methods. This study demonstrates these methods as a successful proof of concept for evaluation of medical education courses, in particular Massively Open Online Courses (MOOCs).

In the context of the ongoing COVID-19 pandemic, where many universities are closed and plans are in place to re-start learning activities in virtual formats [42, 43], these courses are of increasing importance for the foreseeable future as will virtual methods of evaluation.

Previous literature has established e-learning as having equal efficacy and satisfaction [1, 5, 8–10, 44] to face to face methods, at lower cost [45] to both under -[46] and post-graduates in medicine [11]. These courses have covered basic science topics such as anatomy [47], but also evidence-based medicine [11], clinical skills [48, 49] and simulation [50]. No literature is available evaluating e-learning for investigators in clinical studies.

Quality assurance of research studies is typically achieved by site initiation visits, study of detailed protocols and accreditation of investigators. As rapid, one-off events, it may be difficult for investigators to retain relevant information and apply protocols appropriately based on these methods. E-learning may provide a more accessible way of disseminating this information, which may be digested at a researcher’s own pace and referred to on demand. These assurance visits are typically only performed for prospective interventional studies, here, the value for observational studies is shown as well.

### Educational evaluation by machine learning methods

Machine learning methods have been implemented in other areas of education research [51] and are used commercially in search engines and social media [20, 52]. Data mining has been used in educational studies generally (not medical education) to gather overall sentiment [16–18] and topic modelling [19] as done in the present study, but also for ‘student modelling’ whereby students’ predicted preferences for teaching and course outcomes are modelled [53–55]. In non-medical MOOCs, recent literature has emerged on the use of topic modelling and sentiment analyses on monitoring feedback and discussions [56–58]. Marking of essays and other free text coursework has also been demonstrated using similar machine learning techniques as in this study [59].

While there has been no or few similar approaches to analyse feedback in medical education, similar principles have been employed in the nascent field of virtual reality simulation in medicine to analyse competence in procedures [60]. Hajshirmohammadi showed their fuzzy model could accurately classify experienced and novice surgeons in knot tying tasks on a virtual reality laparoscopic surgical simulator [61]. Megali [62] and Loukas [63] have further used hidden Markov models and multivariate autoregressive models for the same purpose with increasing success. These methods may pave way for more objective assessment of technical skills in clinical practice.

### Strengths and limitations

Prior studies of e-learning in medicine have thus far have been on a small scale, with only a minority having greater than 100 participants; with inclusion of over 1000 responses, this study has been able to apply machine learning techniques to this larger natural language dataset. Through these machine learning algorithms it is demonstrated a method for reproducibility and objectivity in the analysis of free text data [64, 65]. This study is limited however, in not collecting data on demographic characteristics of feedback respondents. It is thus not possible to determine if respondents are representative of all collaborators who took part in the study, however, since a large majority responded it is unlikely sample was excessively skewed. Further, it not possible to evaluate the effects of demographic factors such as gender, stage of training and country on feedback, which might otherwise facilitate iterative and targeted improvements in the resource content. Although collaborators were required to have a basic level of English proficiency to complete the e-learning course and free text feedback was mandated to be in English, it is possible that non-native speakers may have been dissuaded from entering feedback or refrain from entering as rich feedback as they might in their native language. This effect is also minimised somewhat as the majority of course participants were from English speaking countries.

Conventional qualitative methods such as interviews and focus groups may have yielded a greater depth of information and may have facilitated better exploration of ideas than electronic feedback, face to face visits may also be effective in building relationships, fostering morale and answering specific questions more effectively than e-learning. However, in the present context, these would be impractical due to the large scale of the study, current social distancing guidelines and the geographic distribution of participants. Future directions of research should evaluate e-learning as part of training for randomised controlled trials alongside more traditional methods. Further validation of natural language processing and machine learning approaches to free-text data in medical education are needed.

## Conclusion

E-learning can be a successful method for training participants of large-scale clinical studies and medical students, with high user satisfaction. Natural language processing approaches may be beneficial in evaluating medical education feedback as well as large scale educational programmes, where other methods are impractical and less reproducible.

## Supplementary Information

**Additional file 1.**

**Additional file 2.**

## Data Availability

The e-learning course and feedback questionnaire are available publicly at https://starsurg.org/imagine-e-learning/. The datasets used and/or analysed during the current study available from the corresponding author on reasonable request. Data analysis scripts and a guide on implementing the methods employed are available in supplementary file 1.
