# Supplementary material for: Evaluation of an international medical E-learning course with natural language processing and machine learning
Source: BMC Med Educ. 2021 Mar 25;21:181. doi: 10.1186/s12909-021-02609-8 (PMC7992837; doi:10.1186/s12909-021-02609-8)
Supplement: Supplementary file 1 — Additional file 1. [file 12909_2021_2609_MOESM1_ESM.pdf]

Supplementary File 1 - Analysis and Guide to Implement Methods

Dr Aditya Borakati

Royal Free Hospital, Pond Street, London, NW3 2QG
a.borakati@doctors.org.uk

2021-01-17

1 Preface 5
2 Software Environment 7
3 Load Libraries 9
3.0.1 Loads required packages for analysis: 9
4 Load Data and Data Cleaning 11
5 Bar plot of ratings with mean 13
6 Qualitative Analysis 15
7 Sentiment Analysis 19
7.0.1 AFINN analysis 19
7.0.2 Syuzhet analysis 19
7.0.3 SentimentR 20
8 Topic Modelling 21
9 Model Diagnostics 23
9.1 Log Likelihood: 23
9.2 Perplexity 23
9.3 Multiple Diagnostic Values: 24
9.4 Plot 25

1 Preface

This file serves two purposes:

1. To provide a transparent record for the analysis conducted in the paper 'Evaluation of online training for international, multi-centre collaborative studies: A Qualitative Analysis with Natural Language Processing and Machine Learning Techniques' and enable reproducible research
2. To provide a guide for researchers and educationalists to implement the techniques discussed in their own work, whether that be in surveys of any kind (not just educational), focus groups and indeed any form of free text data

This analysis was implemented in the R programming language (https://mran.microsoft.com/open), however Python (https://www.python.org/), Julia (https://www.julialang.org/) and more offer robust tools to conduct the same analyses. A basic understanding of R is required to follow the code and the author does not attempt to teach this, however recommends the following courses to learn R:

1. HealthyR (https://healthyr.surgicalinformatics.org/) by the University of Edinburgh, available in both online and face to face formats

2. R Programming (https://www.coursera.org/learn/r-programming), a MOOC by Johns Hopkins University

This analysis was further performed using Microsoft Open R, rather than the standard distribution of R from the R Foundation (https://www.r-project.org/foundation/). This is because Microsoft's distribution incorporates Intel's Math Kernel Library (https://software.intel.com/content/www/us/en/develop/tools/math-kernel-library.html) which significantly enhances the performance of many of the machine learning algorithms used here on Intel processors. For this reason, it also recommended to run these analyses using Intel hardware. Running this code takes roughly 1 and a half hours on a modern laptop (with Intel 8550u processor). Smaller datasets with desktop computers or server clusters and graphics processors may be significantly faster.

A HTML version of this document is available here:
(https://aborakati.github.io/E-learning-Analysis/)

2 Software Environment

```
R version 3.5.3 (2019-03-11)
Platform: x86_64-w64-mingw32/x64 (64-bit)
Running under: Windows 10 x64 (build 17763)

Matrix products: default

locale:
[1] LC_COLLATE=English_United Kingdom.1252  LC_CTYPE=English_United Kingdom.1252
[3] LC_MONETARY=English_United Kingdom.1252 LC_NUMERIC=C
[5] LC_TIME=English_United Kingdom.1252

attached base packages:
[1] grid      stats     graphics  grDevices utils     datasets  methods   base

other attached packages:
 [1] tidytext_0.2.0      broom_0.5.2         ldatuning_0.2.0
 [4] sentimentr_2.7.1    syuzhet_1.0.4       vcdExtra_0.7-1
 [7] gnm_1.1-0           vcd_1.4-4           wordcloud_2.6
[10] RColorBrewer_1.1-2  tm_0.7-6            NLP_0.2-0
[13] ggpubr_0.2.3        magrittr_1.5        forcats_0.4.0
[16] stringr_1.4.0       dplyr_0.8.3         purrr_0.3.2
[19] readr_1.3.1         tidyr_1.0.0         tibble_2.1.3
[22] ggplot2_3.2.1       tidyverse_1.2.1     topicmodels_0.2-8
[25] RevoUtils_11.0.3    RevoUtilsMath_11.0.0 topicdoc_0.1.0

loaded via a namespace (and not attached):
 [1] httr_1.4.1        jsonlite_1.6      modelr_0.1.5      assertthat_0.2.1
 [5] stats4_3.5.3      cellranger_1.1.0  slam_0.1-45       pillar_1.4.2
 [9] backports_1.1.4   lattice_0.20-38   glue_1.3.1        ggsignif_0.6.0
[13] rvest_0.3.4       colorspace_1.4-1  Matrix_1.2-15     pkgconfig_2.0.2
[17] qdapRegex_0.7.2   haven_2.1.1       scales_1.0.0      generics_0.0.2
[21] relimp_1.0-5      withr_2.1.2       nnet_7.3-12       lazyeval_0.2.2
[25] cli_1.1.0         crayon_1.3.4      readxl_1.3.1      tokenizers_0.2.1
[29] janeaustenr_0.1.5 SnowballC_0.6.0   nlme_3.1-137      MASS_7.3-51.1
[33] xml2_1.2.2        tools_3.5.3       data.table_1.12.2 hms_0.5.1
[37] lifecycle_0.1.0   munsell_0.5.0     compiler_3.5.3    lexicon_1.2.1
[41] ca_0.71           rlang_0.4.0       rstudioapi_0.10   qvcalc_0.9-1
[45] gtable_0.3.0      R6_2.4.0          zoo_1.8-6         lubridate_1.7.4
[49] textclean_0.9.3   zeallot_0.1.0     modeltools_0.2-22 stringi_1.4.3
[53] parallel_3.5.3    Rcpp_1.0.2        vctrs_0.2.0       tidyselect_0.2.5
[57] lmtest_0.9-36
```

3 Load Libraries

3.0.1 Loads required packages for analysis:

```
library(tidyverse)
library(ggpubr)
library(tm)
library(wordcloud)
library(vcdExtra)
library(syuzhet)
library(sentimentr)
library(topicmodels)
library(ldatuning)
library(tidytext)
library(topicdoc)
```

4 Load Data and Data Cleaning

```
data              <- read_csv
("/
  E-learning Data/E-learning Feedback (Responses) - Form Responses 1.csv",
                           col_types = cols(`How would you rate the e-learning overall?` =
         col_integer()))
```

Change variable names:

```
# ##Ratings has a numerical rating for
# the course overall from 1 5 (5 being
# highest)

ratings <- table(data$`How would you rate the e-learning overall?`)

# ##Good has freetext responses for the
# question below:

good <- data$`What was good about the e-learning overall?`

# ##Bad has freetext responses for the
# question below:

bad <- data$`What could be improved about the e-learning overall?`

# ##Other has freetext responses for the
# question below:

other <- data$"Any other comments about the e-learning overall:"
```

Mean and standard deviation of overall ratings:

```
meanrating <- mean(data$`How would you rate the e-learning overall?`)
sdrating <- sd(data$`How would you rate the e-learning overall?`)
```

Normality testing:

```
gghistogram(ratings, xlab = "Ratings")
ggqqplot(ratings)
shapiro.test(ratings)
```

All show non normal distribution (expected)

5 Bar plot of ratings with mean

This generates a bar chart with the number of responses for the question 'How would you rate the e learning overall?', in each category of 1 5, with a vertical line where the mean rating is

```
ggplot(data, aes(x = factor(data$`How would you rate the e-learning overall?`))) +
    geom_bar(stat = "count", width = 0.7,
        fill = "steelblue") + geom_vline(xintercept = 4.56,
    color = "orange", size = 2) + coord_cartesian(clip = "off") +
    geom_text(stat = "count", aes(label = ..count..),
        vjust = -0.5, color = "black", size = 3.5) +
    geom_text(x = 4.56, y = -55, label = "Mean = 4.56",
        size = 3, colour = "black") + ggtitle("Overall Rating of E-learning Course by
         Participants") +
    xlab("Ratings (1-5/5)") + ylab("Number") +
    theme_minimal() + theme(plot.title = element_text(hjust = 0.5))
```

This corresponds to Figure 2:

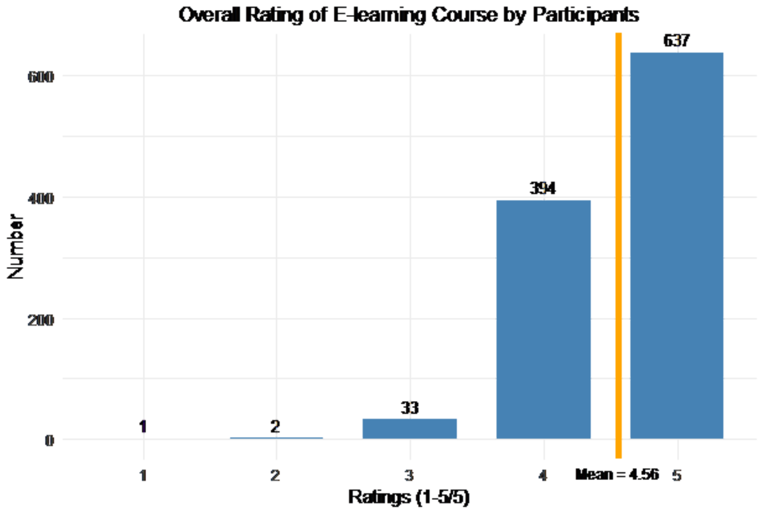

6 Qualitative Analysis

Converts 'good' into an object of type 'corpus' for the tm package (for text mining analysis)

```
goodcorpus <- Corpus(VectorSource(good))
```

This code removes extraneous text which may be analysed by the package e.g. 'the', 'a', punctuation, converts to lower case, so upper and lower case words aren't treated seperately, removes whitespace which are counted separately

```
# ##Convert all to lower case
goodcorpus <- tm_map(goodcorpus, content_transformer(tolower))

# ##Remove English 'stopwords' e.g. a,
# the,
goodcorpus <- tm_map(goodcorpus, removeWords,
    stopwords("english"))
goodcorpus <- tm_map(goodcorpus, removeWords,
    c("the"))

# ##Remove Punctuation
goodcorpus <- tm_map(goodcorpus, removePunctuation)

# ##Remove whitespace
goodcorpus <- tm_map(goodcorpus, stripWhitespace)
```

This code creates a 'Term Document Matrix' for the 'goodcorpus', this is a table of each word that appears, with the frequency of each word

```
matrix <- TermDocumentMatrix(goodcorpus)
```

The following code sorts the matrix in decreasing order of frequency:

```
m <- as.matrix(matrix)
v <- sort(rowSums(m), decreasing = TRUE)
d <- data.frame(word = names(v), freq = v)
```

This generates the values for table 3 in the manuscript 'Frequency of top 20 words entered in response to question 'What was good about the e learning overall?'

The following code generates a wordcloud, with the size of word proportional to it's frequency in the 'goodcorpus'

```
wordcloud(words = d$word, freq = d$freq,
    min.freq = 1, max.words = 20, random.order = FALSE,
    rot.per = 0.35, colors = brewer.pal(8,
        "Dark2"))
```

The rest of the code in this section generates a TermDocumentMatrix and wordcloud as above but for the 'badcorpus' and 'othercorpus'

```
# Bad feedback
badcorpus <- Corpus(VectorSource(bad))

# Transform to remove extraneous text
# e.g. 'the' punctuation, convert to
# lower case
badcorpus <- tm_map(badcorpus, content_transformer(tolower))
badcorpus <- tm_map(badcorpus, removeWords,
    stopwords("english"))
badcorpus <- tm_map(badcorpus, removeWords,
    c("the"))
badcorpus <- tm_map(badcorpus, removePunctuation)
badcorpus <- tm_map(badcorpus, stripWhitespace)
matrix2 <- TermDocumentMatrix(badcorpus)

# Sort matrix by most common words
m2 <- as.matrix(matrix2)
v2 <- sort(rowSums(m2), decreasing = TRUE)
d2 <- data.frame(word = names(v2), freq = v2)

# Wordcloud
wordcloud(words = d2$word, freq = d2$freq,
    min.freq = 1, max.words = 20, random.order = FALSE,
    rot.per = 0.35, colors = brewer.pal(8,
        "Dark2"))

# Other feedback
othercorpus <- Corpus(VectorSource(other))

# Transform to remove extraneous text
# e.g. 'the' punctuation, convert to
# lower case
othercorpus <- tm_map(othercorpus, content_transformer(tolower))
othercorpus <- tm_map(othercorpus, removeWords,
    stopwords("english"))
othercorpus <- tm_map(othercorpus, removeWords,
    c("the"))
othercorpus <- tm_map(othercorpus, removePunctuation)
othercorpus <- tm_map(othercorpus, stripWhitespace)
matrix3 <- TermDocumentMatrix(othercorpus)

# Sort matrix by most common words
m3 <- as.matrix(matrix3)
v3 <- sort(rowSums(m3), decreasing = TRUE)
d3 <- data.frame(word = names(v3), freq = v3)
```

```
# Wordcloud
wordcloud(words = d3$word, freq = d3$freq,
    min.freq = 1, max.words = 20, random.order = FALSE,
    rot.per = 0.35, colors = brewer.pal(8,
        "Dark2"))
```

7 Sentiment Analysis

Merge all 3 corpuses (good, bad and other) into one:

```
combined <- rbind(d, d2, d3)
combinedvector <- rbind(good, bad, other)
combinedcorpus <- Corpus(VectorSource(combinedvector))
```

7.0.1 AFINN analysis

This section takes the AFINN lexicon (a dictionary of the English language with scores assigned depending on how positive or negative the word is judged to be) and assigns that score to each word in the 'combined' corpus

```
afinn <- get_sentiments("afinn")
afinncomb <- inner_join(combined, afinn)
afinncomb <- mutate(afinncomb, sum = freq *
    score)
```

Unweighted mean and standard deviation of setiment scores:

```
meansent <- (sum(afinncomb$sum)/sum(afinncomb$freq))
sd(afinncomb$score)
afcomb <- select(afinncomb, freq, score)
```

Weighted average and standard deviation, weighted by frequency of each word:

```
fr <- expand.table(afcomb, freq = "freq")
mean(fr$score)
sd(fr$score)
```

This generates the afinn score

7.0.2 Syuzhet analysis

This code does the same as the above but for the syuzhet lexicon:

```
syuzhet <- get_sentiment_dictionary(dictionary = "syuzhet")
syuzhetcomb <- inner_join(combined, syuzhet)
syuzhetcomb <- mutate(syuzhetcomb, sum = freq *
```

```
    value)
meansentsz <- (sum(syuzhetcomb$sum)/sum(syuzhetcomb$freq))
sd(syuzhetcomb$score)
szcomb <- select(syuzhetcomb, freq, value)
fs <- expand.table(szcomb, freq = "freq")
mean(fs$value)
sd(fs$value)
```

7.0.3 SentimentR

This code uses the sentimentr package to generate the syuzhet lexicon scores, this takes into account valence shifters e.g. this was not good, which would otherwise be evaluated as a positive for the word good

```
combinedvector <- combinedvector[!is.na(combinedvector)]
syzsent <- sentiment_by(combinedvector)
summary(syzsent)
sd(syzsent$ave_sentiment)
```

These values are used in the manuscript

8 Topic Modelling

Create Document Term Matrix without stopwords for combinedcorpus: *n.b. 'Document Term Matrix' is different to the 'Term Document Matrix' above the former has each word as a column, with frequencies in the row, the latter has each word as a row with frequencies in the column*

```
combinedcorpusrem <- tm_map(combinedcorpus,
    removeWords, stopwords("english"))

combineddtm <- combinedcorpusrem %>% DocumentTermMatrix()

# #Remove empty rows
rowTotals <- apply(combineddtm, 1, sum)
combineddtm <- combineddtm[rowTotals > 0,
    ]
```

This code finds the optimum number of thematic topics by Latent Dirichlet Allocation, for speed of computation, it is run in chunks of 50:

```
result <- FindTopicsNumber(combineddtm, topics = seq(from = 2,
    to = 50, by = 1), metrics = c("Griffiths2004",
    "CaoJuan2009", "Arun2010", "Deveaud2014"),
    method = "Gibbs", control = list(seed = 77),
    mc.cores = 4L, verbose = TRUE)

FindTopicsNumber_plot(result)


result2 <- FindTopicsNumber(combineddtm,
    topics = seq(from = 51, to = 100, by = 1),
    metrics = c("Griffiths2004", "CaoJuan2009",
        "Arun2010", "Deveaud2014"), method = "Gibbs",
    control = list(seed = 77), mc.cores = 4L,
    verbose = TRUE)

FindTopicsNumber_plot(result2)

result3 <- FindTopicsNumber(combineddtm,
    topics = seq(from = 101, to = 150, by = 1),
    metrics = c("Griffiths2004", "CaoJuan2009",
        "Arun2010", "Deveaud2014"), method = "Gibbs",
    control = list(seed = 77), mc.cores = 4L,
    verbose = TRUE)

FindTopicsNumber_plot(result3)
```

The plot of 'result1' generates supplementary figure 1 which shows 6 is the optimum number of topics (greater numbers diverge from the optimum) as evaluated by 4 different methods:

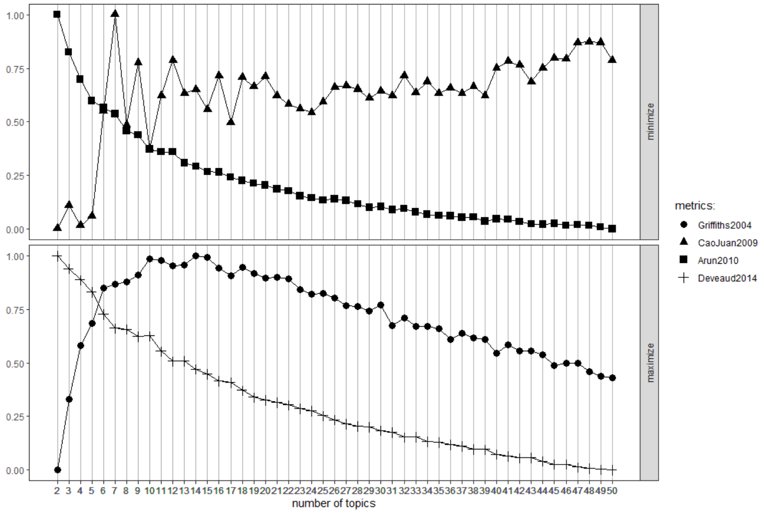

Topic generation, with 6 topics:

```
lda <- LDA(combineddtm, 6, method = "Gibbs")
ldab <- tidy(lda)
ldab <- arrange(ldab, topic, desc(beta))
```

'ldab' is a dataframe which contains each word sorted by topic and frequency (frequency = beta), this gives the results in table 6

9 Model Diagnostics

Please see:

Jordan Boyd-Graber, David Mimno, and David Newman, 2014. Care and Feeding of Topic Models: Problems, Diagnostics, and Improvements. CRC Handbooks of Modern Statistical Methods. CRC Press, Boca Raton, Florida. Available from: [(https://home.cs.colorado.edu/~jbg/docs/2014_book_chapter_care_and_feeding.pdf)](https://home.cs.colorado.edu/~jbg/docs/2014_book_chapter_care_and_feeding.pdf)

For more detailed information on the diagnostic metrics below.

9.1 Log Likelihood:

```
lda@loglikelihood
```

```
[1] -22061.33
```

9.2 Perplexity

```
exp(-1 * (lda@loglikelihood/7122))
```

```
[1] 22.14544
```

The log likelihood and perplexity are both omnibus measures of how well the LDA model predicts the corpus of text. Lower values are better.

9.3 Multiple Diagnostic Values:

```
diag_df <- topic_diagnostics(lda, combineddtm)
diag_df <- diag_df %>% mutate(topic_label = terms(lda,
    5) %>% apply(2, paste, collapse = ", "),
    topic_label = paste(topic_num, topic_label,
        sep = " - "))

diag_df
```

Topic Number	Topic Size	Mean Token Length	Distance from Corpus	Hellinger Distance from Token to Document	Prominence	Coherence	Exclusivity	5 Most Frequent Words
1	206.288	5.8	0.526838	0.110258	24	-206.429	9.696937	1 - easy, understand, good, follow, end
2	184.6986	5.8	0.499784	0	19	-231.868	9.594147	2 - clear, easy, use, simple, useful
3	191.0681	6.6	0.533508	0.108121	13	-208.624	9.632935	3 - questions, very, well, short, really
4	202.9427	7.4	0.538766	0.114748	16	-219.931	9.714171	4 - nothing, clear, concise, module, examples
5	207.9337	5.1	0.554422	0.092861	12	-210.759	9.904656	5 - more, informative, nil, test, none
6	194.0688	5.7	0.545239	0.140479	24	-195.376	9.852739	6 - good, information, the, data, redcap

9.3.0.1 See below for basic description of metrics

9.4 Plot

9.4.0.1 Generate bart charts of above metrics:

```
diag_df %>% gather(diagnostic, value, -topic_label,
    -topic_num) %>% ggplot(aes(x = topic_num,
    y = value, fill = str_wrap(topic_label,
        25))) + geom_bar(stat = "identity") +
    facet_wrap(~diagnostic, scales = "free") +
    labs(x = "Topic Number", y = "Diagnostic Value",
        fill = "Topic Label", title = "All Topic Model Diagnostics")
```

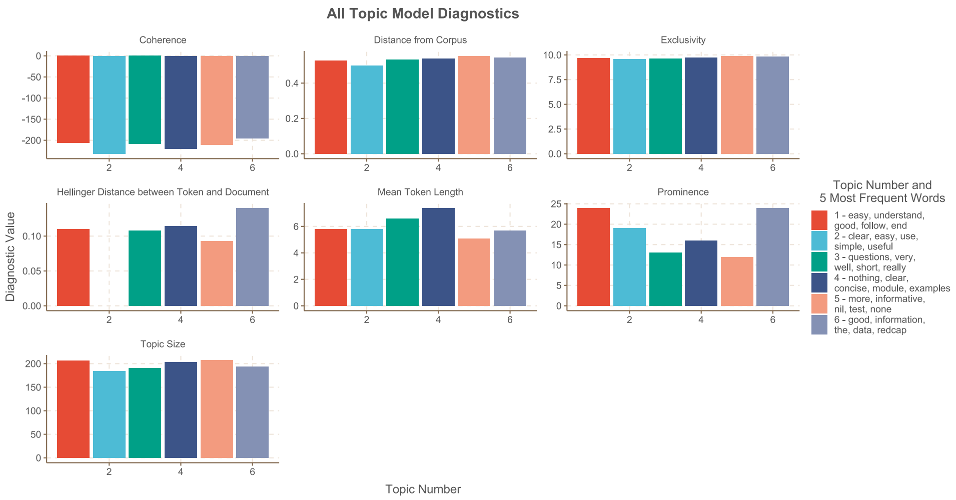

Coherence- Measure of how similar the words in a defined topic are in terms of meaning (or semantics), larger values are better; the measure used here is as described in:

Mimno, D., Wallach, H. M., Talley, E., Leenders, M., & McCallum, A. (2011, July). "Optimizing semantic coherence in topic models." In Proceedings of the Conference on Empirical Methods in Natural Language Processing (pp. 262-272). Association for Computational Linguistics. Chicago

Distance from Corpus- This is the Hellinger distance of the average probability of the words in each topic from the average probability distribution of the same words across the entire corpus

Exclusivity- Measure of how distinct top 10 words in each topic is from other topics see:

Bischof, Jonathan, and Edoardo Airoldi. 2012. “Summarizing topical content with word frequency and exclusivity.” In Proceedings of the 29th International Conference on Machine Learning (ICML12), eds John Langford and Joelle Pineau.New York, NY: Omnipress, 201–208.

Prominence- Number of Words in each topic with a probability of >0.2 in the overall corpus

Mean Token Length- Mean number of characters in each word

Topic Size- No of unique words in each topic
