# Supplementary material for: Evaluation of an international medical E-learning course with natural language processing and machine learning
Source: BMC Med Educ. 2021 Mar 25;21:181. doi: 10.1186/s12909-021-02609-8 (PMC7992837; doi:10.1186/s12909-021-02609-8)
Supplement: Supplementary file 2 — Additional file 2. [file 12909_2021_2609_MOESM2_ESM.docx]

### Supplemental file 2- Feedback questionnaire items; *indicates mandatory question; questionnaire accessible at: <https://starsurg.org/imagine-e-learning/> (no longer taking responses)

| Question | Response options |
| --- | --- |
| **(1) How would you rate the e-learning overall?*** | Very bad 1 2 3 4 5 Very good |
| **(2) What was good about the e-learning overall?** | Free text |
| **(3) What could be improved about the e-learning overall?** | Free text |
| **(4) Any other comments about the e-learning overall?** | Free text |
| **(5) Add any specific comments about module 1 (Inclusion/Exclusion criteria) here:** | Free text |
| **(6) Add any specific comments about module 2 (Gastrointestinal Function) here:** | Free text |
| **(7) Add any specific comments about module 3 (Clavien-Dindo Classification) here:** | Free text |
| **(8) Add any specific comments about module 4 (REDCap and Data Protection) here:** | Free text |
